# Emerged HA and NA Mutants of the Pandemic Influenza H1N1 Viruses with Increasing Epidemiological Significance in Taipei and Kaohsiung, Taiwan, 2009–10

**DOI:** 10.1371/journal.pone.0031162

**Published:** 2012-02-06

**Authors:** Chuan-Liang Kao, Ta-Chien Chan, Chu-Han Tsai, Kuan-Ying Chu, Shu-Fang Chuang, Chang-Chun Lee, Zheng-Rong Tiger Li, Ko-Wen Wu, Luan-Yin Chang, Yea-Huei Shen, Li-Min Huang, Ping-Ing Lee, ChingLai Yang, Richard Compans, Barry T. Rouse, Chwan-Chuen King

**Affiliations:** 1 Institute of Epidemiology and Preventive Medicine, College of Public Health, National Taiwan University (NTU), Taipei, Taiwan, Republic of China (ROC); 2 Department of Clinical Laboratory Sciences & Medical Biotechnology, College of Medicine, NTU, Taipei, Taiwan, Republic of China (ROC); 3 Department of Laboratory Medicine, NTU Hospital, Taipei, Taiwan, Republic of China (ROC); 4 Department of Pediatrics, NTU Hospital, Taipei, Taiwan, Republic of China (ROC); 5 Institute of Biomedical Informatics, School of Life Sciences, National Yang-Ming University, Taipei, Taiwan, Republic of China (ROC); 6 Department of Internal Medicine, Yuan's General Hospital, Kaohsiung, Taiwan, Republic of China (ROC); 7 Department of Microbiology and Immunology and Emory Vaccine Center, Emory University School of Medicine, Atlanta, Georgia, United States of America; 8 Department of Pathobiology, College of Veterinary Medicine, University of Tennessee, Knoxville, Tennessee, United States of America; University of Alabama at Birmingham, United States of America

## Abstract

The 2009 influenza pandemic provided an opportunity to observe dynamic changes of the hemagglutinin (HA) and neuraminidase (NA) of pH1N1 strains that spread in two metropolitan areas -Taipei and Kaohsiung. We observed cumulative increases of amino acid substitutions of both HA and NA that were higher in the post–peak than in the pre-peak period of the epidemic. About 14.94% and 3.44% of 174 isolates had one and two amino acids changes, respective, in the four antigenic sites. One unique adaptive mutation of HA2 (E374K) was first detected three weeks before the epidemic peak. This mutation evolved through the epidemic, and finally emerged as the major circulated strain, with significantly higher frequency in the post-peak period than in the pre-peak (64.65% vs 9.28%, p<0.0001). E374K persisted until ten months post-nationwide vaccination without further antigenic changes (e.g. prior to the highest selective pressure). In public health measures, the epidemic peaked at seven weeks after oseltamivir treatment was initiated. The emerging E374K mutants spread before the first peak of school class suspension, extended their survival in high-density population areas before vaccination, dominated in the second wave of class suspension, and were fixed as herd immunity developed. The tempo-spatial spreading of E374K mutants was more concentrated during the post–peak (p = 0.000004) in seven districts with higher spatial clusters (p<0.001). This is the first study examining viral changes during the naïve phase of a pandemic of influenza through integrated virological/serological/clinical surveillance, tempo-spatial analysis, and intervention policies. The vaccination increased the percentage of E374K mutants (22.86% vs 72.34%, p<0.001) and significantly elevated the frequency of mutations in Sa antigenic site (2.36% vs 23.40%, p<0.001). Future pre-vaccination public health efforts should monitor amino acids of HA and NA of pandemic influenza viruses isolated at exponential and peak phases in areas with high cluster cases.

## Introduction

Newly emerged triple reassortant 2009 pandemic influenza A (HIN1) (pH1N1) viruses were detected in patients with respiratory illness in Mexico and the United States in early April, 2009 [1]–[3]. These novel viruses rapidly spread worldwide through human-to-human transmission. The World Health Organization (WHO) announced its pandemic alert levels as phase 4–5 in late April, 2009, elevated to phase 6 on June 11, 2009, and then moved to the post-pandemic period on August 10, 2010. As of August 1, 2010, more than 214 countries and overseas territories had reported laboratory-confirmed cases of pH1N1, including over 18449 deaths (http://www.who.int/csr/don/2010_08_06/en/index.html). How these novel influenza viruses underwent spontaneous evolution [4], [5] and dynamic changes over different time periods and various places within different epidemiological entities and intervention strategies is an important public health issue.

Hemagglutinin (HA) and neuraminidase (NA) are the two most important envelope proteins of influenza viruses, frequently encountering external antibody selection pressure. HA, a major surface glycoprotein of influenza virus responsible for its attachment to host cells and initiating viral entry, plays a significant role in inter-species transmission, emergence of novel influenza viruses, viral pathogenesis and immunity [6], [7]. The NA is involved not only in progeny virion release and spread of the virus from infected cells to their neighboring cells but also may have a role in HA-mediated membrane fusion and assisting efficient replication of influenza viruses [8]–[10]. In addition, the increased levels of accumulated antibodies against HA and NA at the population level also facilitate influenza virus to undergo antigenic drifts under the selective pressure of herd immunity [11]–[13]. After the introduction of pH1N1 viruses into the human population, their variations in the amino acids of HA and NA proteins have been reported [14]–[19]; however these studies lacked important epidemiological attributes, including temporal and geographical comparisons and with or without public health prevention efforts such as school suspension and vaccination. Therefore, the impact of amino acids changes of HA and NA of these pH1N1 viruses related to epidemiological characteristics, clinical severity, and after public health interventions has remained unclear.

Taiwan, with a population density of 639/km^2^, is located at the junction between subtropical and tropical regions of South-East Asia. The two metropolitan cities, Taipei and Kaohsiung situated in northern and southern Taiwan, respectively, have much higher population densities (9593/km^2^ and 9948/km^2^) [20]. The first imported case of pH1N1 in Taiwan was identified on May 20, 2009 and the first indigenous pH1N1 case was confirmed five days later [21]. The community outbreak occurred in early July, 2009. Then, the pH1N1 quickly swept through the whole Taiwan and the first fatal case was reported on July 30, 2009 [21]. As of July 31, 2010, a total of 46 fatal cases infected with the pH1N1 were laboratory-confirmed in Taiwan (http://flu.cdc.gov.tw/public/Data/0841041371.pdf). For controlling the spread of pH1N1 in Taiwan, a 2-3-5 class suspension intervention was implemented on September 1 (weeks 35), 2009 for all students who were 18 years old or younger with suspected influenza like illness (ILI) or a positive rapid influenza A antigen test [22]. Under the 2-3-5 policy, any school class must be suspended if more than 2 students were ILI cases (fitting the above criteria) within a time interval of 3 days. Students from suspended class were required to stay home for at least 5 days before their return to school. A pH1N1-specific mass vaccination program targeted at elementary through junior high school students free of charge began on November 16, 2009 [23]. A nationwide vaccination program was later launched on December 12, 2009. As of July 25, 2010, the overall vaccine coverage rates were 24.6% in the general population and 76.9% among targeted schoolchildren. After the circulation of this novel pH1N1 through a series of transmission chains among human populations in the two metropolitan cities with high population densities, Taipei and Kaohsiung, we were interested to determine whether dynamic changes of amino acids residues of HA and NA occurred at different epidemic periods in 2009–2010.

The specific aims of this study were: (1) to examine the variations in nucleotide and amino acid sequences and co-mutations of HA and NA of the pH1N1 viruses isolated in the post-peak period versus in the pre-peak period of the epidemic in Taipei and Kaohsiung from June 2009 through October 2010, (2) to compare viral mutation rates and qualitative amino acid changes at receptor-binding sites (RBS), antigenic sites and N-glycosylation sites before and after the implementation of three public health interventions, including anti-viral program, school class suspension and vaccination, and (3) to analyze the epidemiologically tempo-spatial conditions in Taipei City that are associated with the spread of the unique mutant of HA of pH1N1 (gradually becoming the dominant strains after the peak of epidemic).

## Materials and Methods

### Study Design and Study Populations

Since influenza is transmitted human-to-human, we initiated virological surveillance by selecting one hospital from each metropolitan located in northern and southern Taiwan, respectively. A cross-sectional study design targeting laboratory-confirmed influenza patients with mild or severe clinical manifestations was implemented. Patients with influenza like illness (ILI) or ILI with severe complications involving any one of the following clinical manifestations within four weeks requiring hospitalizations were recruited for the study: pulmonary complications, neurological complications, myocarditis or pericariditis, invasive bacterial infection or intensive care unit admission (http://www.cdc.gov.tw/ct.asp?xItem=7500&ctNode=920&mp=5) from the National Taiwan University Hospital (NTUH) in Taipei (northern Taiwan, subtropical climate) and Yuan's General Hospital (YGH) in Kaohsiung (southern Taiwan, tropical climate) from June 1, 2009 through October 31, 2010. Their nasopharyngeal or throat swabs were collected for isolation of the pH1N1 viruses and subsequent virological investigation. This research project was approved by the Research Ethics Committee, Institutional Review Board of National Taiwan University Hospital (IRB #200911013R) and the Ethics Committee of Yuan's General Hospital (IRB #2009110501B). Subjects enrolled in this study provided written informed consent for collecting clinical samples for virological testing. All the data analyses of isolated influenza virus strains, clinical and epidemiological information were processed and operated anonymously.

### Laboratory Tests

#### 1. Isolation of pH1N1 viruses

To obtain more isolates of pH1N1, we used Madin-Darby Canine Kidney cells (MDCK) (CCL34: American Type Culture Collection, Rockville, MD, USA) to culture the virus and subsequently confirmed the results with reverse transcription-polymerase chain reaction (RT-PCR) at NTUH in Taipei. In Kaohsiung, specimens were first screened with rapid influenza virus antigen screening (Genzyme Diagnostics, USA) and the positive samples were then confirmed with RT-PCR during the study period to save manpower expenses at a regional hospital (YGH). All the positive pH1N1 viruses had one passage in MDCK cells and were stored at −80°C for further use.

#### 2. Hemagglutination inhibition (HI) test

Human serum samples were tested for their antibody responses against the pH1N1 viruses by HI assay. The HI procedure was performed according to the protocol used by the Centers for Disease Control and Prevention in the United States (US-CDC). Briefly, the human tested serum specimens were pre-treated with receptor destroying enzyme (Denka Seiken Co., Ltd, cat # 370013) to remove non-specific inhibitors. HI assays were performed in V-bottom 96-well microtiter plates with 0.5% turkey's erythrocytes. HI serotiter was defined as the highest dilution of the tested serum sample showing complete inhibition of hemagglutination of 4 units of HA of the pH1N1.

#### 3. Micro-Neutralization (MNt) assay

The MNt assay used the laboratory protocol from the US-CDC with slight modification. Briefly, human serum samples were heat inactivated at 56°C for 30 minutes, and were prepared using two-fold serial dilutions in a 50-µL volume of virus diluent (Eagles' MEM) in 96-well microtiter plates with flat bottom. The diluted sera were then mixed with an equal volume of virus diluent containing the tested pH1N1 influenza virus strain at 2×10^2^ tissue culture infectious doses with 50% cytopathic effect (TCID)_50_/mL. Four control wells of virus plus virus diluent (VC) and virus diluent alone as cell control (CC) were included on each plate. After incubation at 37°C in a 5% CO_2_ humidified atmosphere for one hour, 100 µL of MDCK cells at 1.5×10^4^/mL were added to each well. The plates were incubated at 37°C, 5% CO_2_ for 18–20 hrs. The monolayers were then washed with phosphate buffered saline (0.01 M PBS, pH 7.2) and fixed in cold 80% acetone in PBS for 10 minutes. The presence of viral proteins was detected by enzyme-linked immunosorbent assay at room temperature with a monoclonal antibody against the NP of influenza A (MAB 8257 and 8258, Millipore, MA, USA). The horseradish peroxidase-labeled goat anti-mouse immunoglobulin G (IgG) (Cat #074-1802, Kirkegaard & Perry, Gaithersburg, MD, USA) was used as a secondary antibody. After color formation in the final step, the absorbance was measured at 490 nm (*A*490) The average *A*490 was determined for quadruplicate wells of virus-infected (VC) and mock-infected (CC) control wells, and a neutralizing endpoint was determined by using a 50% inhibition of the infected virus specific signal. The endpoint titer_50_ was expressed as the reciprocal of the highest dilution of serum with *A*490 value less than *X*, where X = [(mean *A*490 of VC wells)−(mean *A*490 of CC wells)]/2 + (mean *A*490 of CC wells).

#### 4. Nucleotide sequencing of HA and NA genes of pH1N1 viruses

Viral RNA was extracted with the Qiagen RNA mini kit (Qiagen, Germany). The HA domain of the HA gene was amplified by one step RT-PCR (SuperScript™ One-Step RT-PCR with Platinum Taq, Invitrogen, Life Technologies, CA, USA). Primer, Uni-12 (5′-AGC AAA AGC AGG-3′) was used for cDNA synthesis [24]. Two primer pairs were used for HA gene amplification: (1) HA-uniF (5′-AGC AAA AGC AGG GGA AAA-3′), HA-R1 (935-5′-ATA TTC TGA AAT GGG AGG CTG GTG-3′), and (2) HA-F2 (605-5′-GTG CTG ACC AAC AAA GTC TC-3′) HA-uniR (5′-AGT AGA AAC AAG GGT GTT TT-3′). Another two primer pairs were used for HA genome sequencing: (1) HA-SF1 (41-5′-CCG CAA ATG CAG ACA CAT TA-3′), HA-SR1 (759-5′-CGG CTC TAC TAG TGT CCA G-3′) and (2) HA-SF2 (678-5′-GTT CAA GCC GGA AAT AGC-3′), HA-SR2 (1674-5′-CCC ATT AGA GCA CAT CCA GAA-3′). In order to analyze the residue change of 374 located at stalk region of HA2, partial genome (nucleotides, 996–1298) was sequenced by using primers: 996F (996-5′- CAC AGG ATT GAG GAA T-3′), 1298R (1298- 5′-CAG GAA ACC ATC ATC AAC-3′) and 1053F (1053-5′-CGG TTT CAT TGA AGG GGG-3′).

The first reverse transcription from RNA to cDNA was performed at 50°C by incubation for 50 minutes; the PCR reaction started by denaturing at 94°C for 6 minutes, and amplified in 40 cycles (ABI 2700, Applied Biosystems, CA, USA) under the following conditions, 94°C for 30 seconds(s); 53°C for 30 s, 72°C for 80 s and a final extension at 72°C for 10 minutes. The PCR products were analyzed by electrophoresis in a 2% agarose gel and visualized by staining the gels with ethidium bromide. They were further purified and sequenced using an ABI Model 3730XL DNA analyzer with ABI Terminator Cycle Sequencing Ready Reaction Kit, V3.1 (Applied Biosystems, CA, USA). Nucleotide sequence analysis and alignment were performed using the Mega 4.1. For NA nucleotides sequence analysis, the nucleic acid extraction, RT-PCR and sequencing were performed according to the methods used for HA sequences analysis except 51°C used for annealing and different primer sets for amplification and sequencing. Two primer pairs were used for NA gene amplification: (1) NA-uniF (5′-AGC AAA AGC AGG AGT-3′); NA-R1 (893-5′-CCA TGC CAG TTA TCC CTG-3′) and (2) NA-F2 (523-5′-GAG TCA GTC GCT TGG TCA-3′); NA-uniR(5′-AGT AGA AAC AAG GAG TTT TTT-3′). Another two primer pairs were used for NA fragment (1401nucleotides) sequencing: (1) NA-SF2 (721-5′-AAT GAC CGA TGG ACC AAG-3′), NA-SR2 (1332-5′-GGA TAT GCT GCT CCC GCT AG-3′) and (2) NA-SF1 (41-5′-GTA TGA CAA TTG GAA TGG C-3′); NA-SR1 (659-5′-CTC CAA CTC TTG ATA GTG TCT G-3′).

#### 5. Quantitative real-time RT-PCR

Viral RNA was extracted from 200 µL of virus by Lab Turbo Virus Mini kit (Taigen Bioscience Coporation, Taipei, Taiwan) and subjected to one step RT-PCR using Precision one step qRT-PCR MasterMix (Primer Design, Southampton, United Kingdom) and ABI PRISM 7500 Fast Real-Time PCR system (Applied Biosystems, USA). The condition for PCR was started at 95°C for denaturing 8 minutes, then at 95°C, 10 s and 60°C, 31 s for 45 cycles using primers (FluA-F :5′- AAGACCAATCCTGTCACCTCTGA-3′; FluA-R : 5′-CAAAGCGTCTACGCTGCAGTCC-3′) and TaqMan probe (FluA probe: 5′FAM –TTTGTGTTCACGCTCACCG T–3′BBQ). The viral loads in the original samples were measured by using influenza M-gene RNA derived from M-gene carried in plasmid (pGEM-T Easy Vector, # A1360, Promega, USA) as the standard.

### Data Analysis and Statistics Tests

#### 1. Epidemiological data analyses on risk factors

Univariate analyses on factors, including age, gender, population density, residential district, time intervals (weeks) of before, during and after the starting of different vaccination programs targeting at various populations and disease severity that might affect the differences in frequency of amino acid changes between pre-peak and post–peak periods of the epidemic were assessed by Chi-square tests. The important variables including the pre- and post-peak periods of the 2009 epidemic, the phases before and after initiation of vaccination program, gender, age, residential districts with spatial clustering, levels of population density were used to run stepwise multivariate logistic regression analysis.

#### 2. Tempo-spatial analyses and statistic tests

To further analyze the temporal and spatial spread of pH1N1 virus mutants with E374K, we classified eight time stages as maps [(1) Weeks 21–33 (pre-peak and pre-class suspension), (2) weeks 34–35 (the first detection of E374K mutant at week 34, the first week implemented class suspension policy at week 35), (3) week 36, (4) week 37 (peak of epidemic), (5) week 38, (6) week 39, (7) week 40, and (8) weeks 41–52 (week 44+: post-vaccination)], based on the pre-, during and post-epidemic peak periods of epidemic curve in Taipei City and nearby Taipei County. The geographical unit was determined, according to the administrative districts with known population density (person/km^2^) in 2009. The GIS software of ArcGIS (ArcMap, version9.2; ESRI Inc., Redlands, CA, USA) was applied to presenting the emergence and further spatial spread of E374 K mutants in each time period over eight time stages.

In order to examine the spatial clustering of E374K mutants in Taipei City and County, we applied Moran's I to test the presence of global spatial clustering for the different time stages. The local indicators of spatial association (LISA) were used for further identifying local spatial clusters in post-peak epidemic periods stratifying by before versus after the implementation of 1^st^ day of vaccination (21^st^ –43^th^ week vs 44^th^–52^nd^ week) on November 1^st^, 2009, once global autocorrelation spatial showed statistically significant. Global Moran's I was used to evaluate whether the tested districts with clustering tendency, which range from −1 to +1. A reading close to 1 indicates strong spatial autocorrelation and vice versa, with zero indicating randomness. LISA was used to identify where clusters were located. A negative value of LISA reflects dissimilarity with neighbors, while a positive value means similarity, and zero refers to randomness [25].

## Results

### Comparison of the positive detection rates of pH1N1 in northern and southern Taiwan

**Figure 1:**
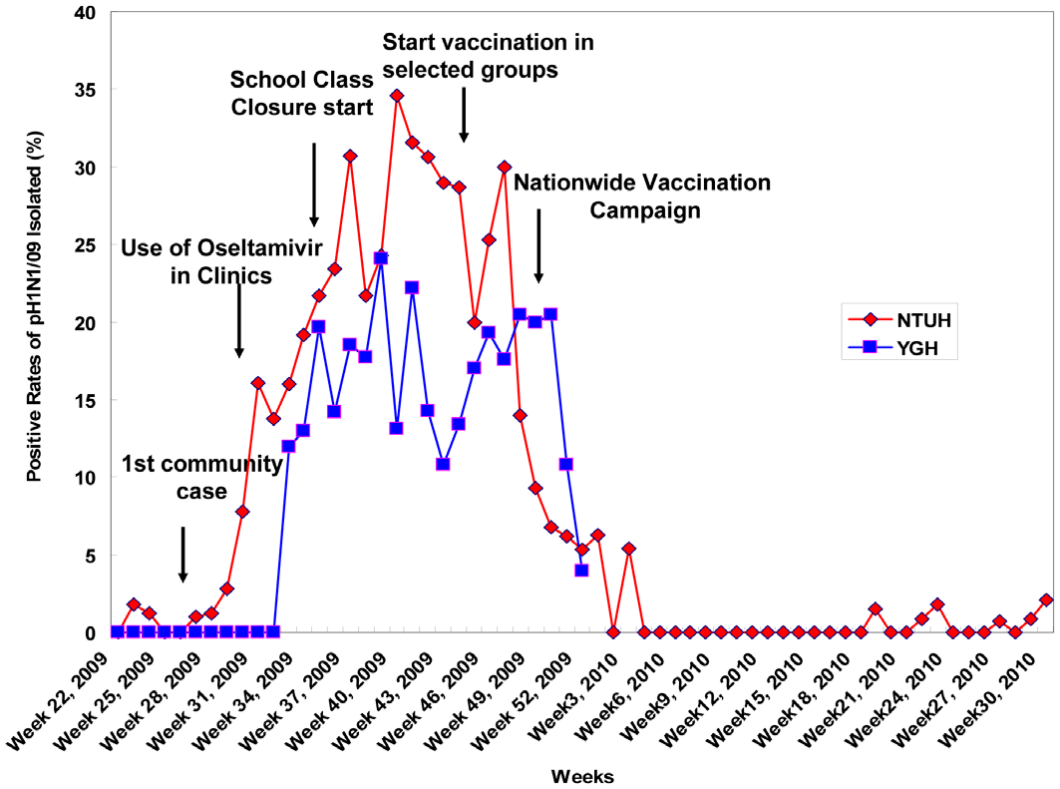
Weekly distributions of positive rates of pH1N1 isolated from NTUH and YGH in Taiwan, 2009–2010. NTUH: National Taiwan University Hospital located in Taipei; YGH: Yuan's General Hospital (YGH) located in Kaohsiung. The time frames of different public health prevention/control measures was indicated with arrows.

In order to monitor molecular changes of pH1N1 virus in the two metropolitan areas, clinical specimens from ILI patients with and without severe complications from June 1, 2009 to October 31, 2010 were collected. The pH1N1 viruses were identified initially by culture or rapid screening tests and finally confirmed by RT-PCR method. The weekly distributions of pH1N1 positive detection rates in Taipei and Kaohsiung during this study period were quite similar (Figure 1). Our analyses on isolation rates of pH1N1 in Taipei and in Kaohsiung were 16.47% (1024/6218) and 16.23% (682/4201) respectively, from week 22, 2009 to week 30, 2010. However, detailed analyses on the isolation rates of pH1N1 in Taipei and in Kaohsiung during the same time period (from week 22, 2009 to week 52, 2009) with the specimens collected for improved comparison were 19.07% (1024/5371) and 16.68% (634/3801), respectively. In other words, the isolation rate in Taipei was higher than that in Kaohsiung though the patterns were similar in both of these metropolitan areas. The first peak in positive rates occurred at the 37th week (September 13–19, 2009) in Taipei and at the 39th week (September 27–October 3, 2009) in Kaohsiung. Furthermore, both the exponential increasing and decreasing phases of the positive pH1N1 rates in Kaohsiung were two weeks later than those in Taipei. Finally, pH1N1 positive rates in these two cities decreased sharply at the end of 2009 and few positive cases were detected sporadically in 2010.

### Analysis of the diversity of amino acid residues and dynamic changes of antigenic sites and receptor-binding sites in HA1 of pH1N1 before and after vaccination

**Figure 2:**
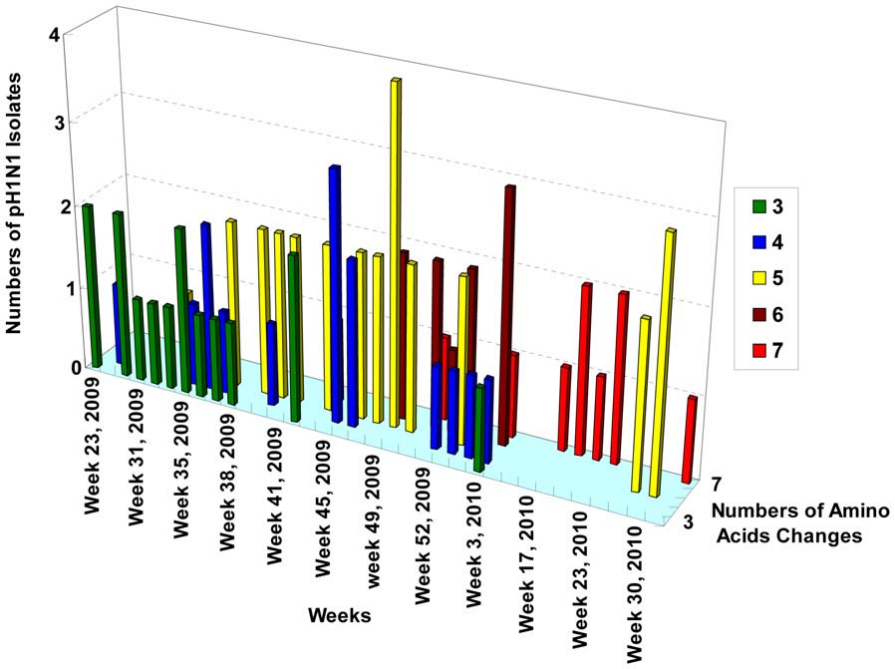
Trends in increasing numbers of amino acids changes of pH1N1-HA in Taiwan, 2009–2010.

The complete HA1 and partial HA2 nucleotide regions (HA1: 52–1029 nucleotides; HA2: 1033–1637 nucleotides) of pH1N1 strains were amplified and sequenced. Using vaccine strains, A/California/07/2009(H1N1) as a reference strain for alignment, 78 available sequenced strains (collected from June 11, 2009 to August 2, 2010, including 50 strains from Taipei and 28 strains from Kaohsiung), showed high conservation, with 99.48% and 99.07% identities in the nucleotides and the amino acid sequences of HA, respectively. The average substitution rates of nucleotides and amino acids for HA were 5.04×10^−3^ per nucleotide per gene and 9.60×10^−3^ per amino acid per protein, respectively. The number of amino acid differences in HA between the Taiwanese isolates and the vaccine strain [A/California/07/2009(H1N1)] ranged from 3 to 7. There was a trend of increasing frequency of cumulative numbers of amino acid substitutions over the weekly time periods (Figure 2). The frequency of pH1N1 with cumulative numbers of amino acids (equal to and greater than 5) was significantly higher in the post–peak period of the epidemic than those pH1N1 isolated at the pre-peak period (74.6%, 47/63 vs 6.67%, 1/15) (p<0.0001, Table 1).

**Table 1:** Comparison of the numbers of amino acid residue changes in hemagglutinin and neuraminidase of Taiwanese pH1N1 viruses between pre-peak and post-peak periods of the pH1N1 epidemic in Taipei and Kaohsiung of Taiwan, 2009–2010.

	Hemagglutinin					Neuraminidase		
Periods of Epidemic Stages and Phases Related to Public Health Efforts	Numbers of isolates	Numbers of amino acid changes**		Fisher's exact p-values	Numbers of isolates	Numbers of amino acid changes**		Fisher's exact p-values
		<5	≧5			<3	≧3	
**A. Pre-peak Period of the Epidemic** * (<Week 37, before Sept. 13, 2009)	15	14 (93.3%)	1 (6.7%)		19	12 (63.2%)	7 (36.8%)	
**B. Post-peak Period of the Epidemic** (≧Week 37, Sept. 13, 2009 and after∼)	63	16 (25.4%)	47 (74,6%)	**<0.0001** ***	21	8 (38.1%)	13 (61.9%)	0.205***
**Vaccination program status**								
**1. Pre-vaccination Phase** (Week 37–43, Sept. 13–Oct. 31, 2009)	17	6 (35.3%)	11 (64.7%)					
**2. During vaccination campaign** (Week 44–50, Nov. 1–Dec 19,2009)	17	5 (29.4%)	12 (70,6%)					
3. **Post –vaccination Phase** (>Week 50, After Dec 20,2009)	29	5 (17.2%)	24 (82.8%)	0.398****				

*: Based on the positive rate detected by RT-PCR or virus culture.

**Compared with reference strain: A/California/07/2009.

***: Pre-peak period vs post peak period.

****:Pre-vaccination vs post-vaccination.

**Figure 3:**
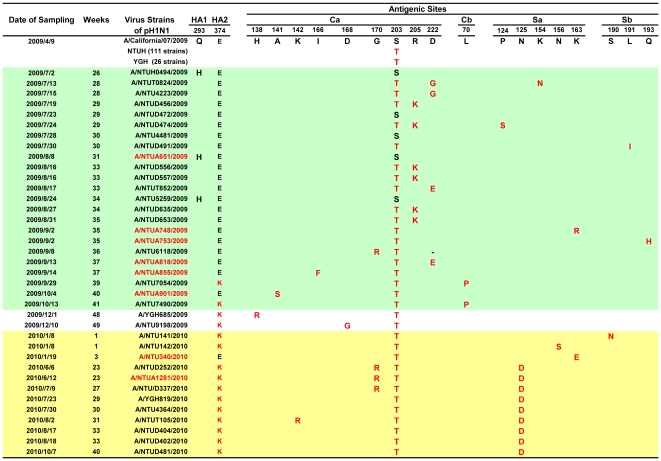
Dynamic changes of amino acid residues at the antigenic sites of pH1N1-HA in Taiwan, 2009–2010. <37th Week: Pre-peak period of the 2009 epidemic; 37th Week: Peak of the 2009 epidemic; >37 ^th^ Week: Post-peak period of the epidemic; Green background: Pre-peak period of the epidemic ; Yellow background: Post-vaccination campaign phase; Strains with red color: pH1N1 strains were isolated from severe cases.

The amino acid residues corresponding to the predicted four antigenic sites Ca, Cb, Sa and Sb located at the globular region (HA1) [26] of the 174 Taiwanese pH1N1 strains [available partial HA nucleotides 261–1029, amino acid 70–343, numbering without signal peptide (17 amino acids)] were compared to the US early isolate (A/California/7/2009). Before August 25, 2009 with most imported cases, only the five wild-type pH1N1 viruses with S203S (13.16%, 5/38) isolated. The S203T, located at antigenic site Ca, was first detected on May 24, 2009 (week 21) and their frequencies were extremely high at 100% (2/2), 78.57% (11/14) and 94.44% (34/36) in June, July and August, 2009, respectively and reached 100% since August 30, 2009 (week 35). These results indicated that 97.13% (169/174) of pH1N1 isolates had amino acid residue substitution of S203T [counting the number from the initial codon without signal peptide] (Figure 3).

**Figure 4:**
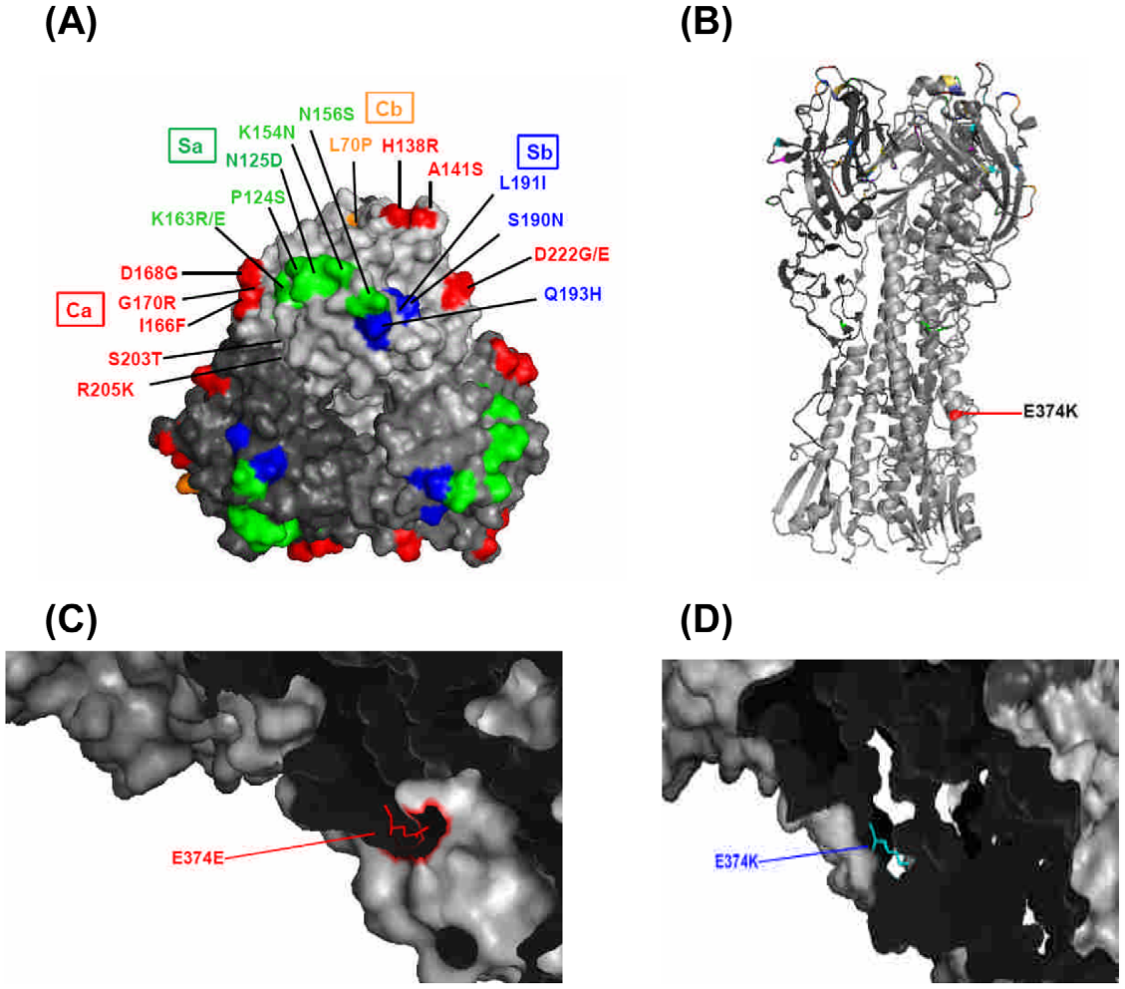
Locations of changed amino acid residues of HA1 and HA2 of pH1N1 virus in Taiwan. **A**. Globular head of HA1. Site Ca (Red); Site Cb Orange); Site Sa (Green); Site Sb (Blue). **B**. Stalk region of HA2. **C**. Early isolate with E374E residue. **D.** Late isolate with E374K. All the figures were generated and rendered with the use of MacPyMOL (http://wwwpymolorg). Numbers of amino acids represents the order of the amino acid taking out signal peptide (17 amino acids) and then counting the numbers from initial codon.

Besides the S203T, the frequencies of pH1N1 with other substitutions as one and two more amino acids changes at all four antigenic sites were much lower as 14.94% (26/174) and 3.44% (6/174), respectively (Figure 3). However, their overall frequency of amino acid substitutions at all four antigenic sites of HA1, with the exception of S203T, was higher in viruses isolated at post-peak period (19.59%, 19/97) than those at pre-peak period (16.88%, 13/77, p = 0.648), although the differences were not statistically significant. The ranking of frequencies of amino acid mutations from the highest to the lowest observed was 19, 14, 3 and 2 at the antigenic sites of Ca, Sa, Sb and Cb, respectively [Figure 3, 4A]. To more clearly describe the dynamic antigenic changes of this pH1N1 related to the timing of vaccination, we divided the post-epidemic time period into three phases: (1) pre-vaccination phase (Weeks 37–43, September 13–October 31, 2009), (2) during the on-going vaccination campaign phase [Weeks 44–50, November 1–December 19 (December 12+7 days for antibody production for evaluating different levels of herd immunity, 2009)], and (3) post-nation-wide vaccination campaign phase (>Week 50, after December 19, 2009). The overall frequencies of pH1N1 viruses with amino acid substitutions in all four antigenic sites in each of the three phases were 13.16% (5/38), 11.76% (2/17) and 28.57% (12/42), respectively [p = 0.149]. All the residue changes at the four antigenic sites from the pre-peak period of the 2009 epidemic through the pre-vaccination phase of the post-peak period of the 2009 epidemic (Figure 3) were examined, including: (1) site Ca [with the most variations involving R205K (6 strains), D222G/E(4 strains), A141S(1 strain), I166F (1 strain)]; (2) site Cb [L70P(2 strains)]; (3) site Sa [P124S (1 strain), K154N(1 strain), K163R(1 strain)]; and (4) site Sb [L191I (1 strain), Q193H (1 strain)] (shown as green zone in Figure 3). Interestingly, all these variants no longer were detected during the post-vaccination phase. However, there were also pH1N1 variants with amino acid substitutions at five locations in the three antigenic sites identified only at post-nation-wide vaccination campaign phase (after December 19, 2009, shown as yellow zone in Figure 3), including site Ca [K142R (1 strain)], site Sa [N125D (9 strains), N156S (1 strain); K163E (1 strain)], site Sb [S190N (1 strain)], that did not appear in pre-vaccination phase. Among these five variants, one new unique pH1N1 variant N125D was most frequently observed (9/42, 21.43%). It was first detected on June 6, 2010 and then continued to circulate afterwards with another 8 strains till the study period ended in October 2010. During the on-going vaccination campaign phase (shown as white zone in the middle part of Figure 3), we observed 2 residue substitutions, H138R and D168G in site Ca, but these disappeared later on without having been completely fixed. Another pH1N1 variant G170R, with the only substitution located at site Ca was present in both pre- and post-vaccination phases, involving: (1) one strain (isolated on September 8, 2009 in 36^th^ wk) at the pre-peak period of the epidemic prior to vaccination and (2) three strains (isolated on June 6, 12 and July 9, 2010) at the post-nation-wide vaccination campaign phase). Mutations were not evident at the Cb site during the on-going and post-vaccination phases (Figure 3). Taken together, the percentages of mutations of Sa sites were significantly higher in post-nation-wide vaccination phase than pre-nation-wide vaccination phase (23.40%, 11/47 vs 2.36%, 3/127, p<0.001).

To further understand the effect of the substitutions of amino acids at the HA1 antigenic sites on the antigenic property of pH1N1, viruses with different substitutions and isolated in different time periods of the epidemic were tested for HI with the six pH1N1 vaccinated human serum samples (ages ranged from 8–12 years old). The results showed no significant differences in the HI serotiters among various pH1N1 viruses with different amino acid substitutions isolated from the early and late periods of the epidemic (Table 2). For example, HI serological titers showed no significant differences between the two strains (A/Taiwan/NTUD252/2010 and A/Taiwan/NTUT105/2010) with double mutants (HA: N125D and E374K) and the other two strains (A/Taiwan/NTU4223/2009 and A/Taiwan/NTU6118/2009) without double mutants and the other substitutions located at Ca site (K142R, G170R, S203T and D222G) as well, using pH1N1 (A/California/07/2009 with HA N125N, E374E) vaccinated human serum. This finding was also confirmed through testing with the reference pH1N1 virus immunized sheep serum (WHO reagent kit supported by US-CDC) (Table 2).

**Table 2:** Serological comparison in the antibody responses reacted with the pH1N1 isolates with various amino acid substitutions at different antigenic sites by hemagglutination inhibition (HI) tests.

Virus antigen	Amino acid changes	HI Serotiters of Anti-pH1N1						
		Six human serum samples						pH1N1 immune sheep serum^2^
		010^1^	020	030	035	053	063	
**A/California/07/2009 (NYMC X-179A)**	**S203S D222D E374E N125N K142K G170G**	640^4^	1280	640	1280	1280	640	2560
**A/Taiwan/NTU4223/2009 (7/1/2009)**	**S203T D222G E374E**	640	640	320	1280	640	320	2560
**A/Taiwan/NTU6118/2009 (9/8/2009)**	**S203T E374E G170R**	320	640	320	640	640	320	5120
**A/Taiwan/NTU025/2010 (1/6/2010)**	**S203T E374K**	640	1280	640	1280	640	640	5120
**A/Taiwan/NTUD252/2010 (6/7/2010)**	**S203T E374K G170R N125D**	640	640	320	1280	640	320	5120
**A/Taiwan/NTUA1281/2010 (6/12/2010)**	**S203T E374K G170R**	640	640	320	1280	640	320	2560
**A/Taiwan/NTUT105/2010 (8/2/2010)**	**S203T E374K N125D K142R**	640	640	320	640	640	320	5120
**Pandemic A/H1N1 control antigen** 3								5120

1 ID numbers of the tested human serum samples.

2 ATCC IRR FR188 and.

3 ATCC IRR FR-187 in the 2010–2011 WHO Influenza Regent kit for diagnosis of influenza virus from the WHO Collaborating Centers for Surveillance, Epidemiology and control of Influenza at the U.S. Centers for Disease Control and Prevention.

4 HI serotiters: The highest dilution of human serum samples showed complete inhibition of 4 HA units of pH1N1 viruses.

Regarding the RBS, only six strains (isolated in Taipei from July 2009 to January 2010) of the 174 sequenced Taiwanese pH1N1 viruses had amino acid residue changes in RBS of pH1N1-HA1 [27], [28]. Four strains had substitutions in 220-loop region (residues 218–225), including two strains with D222G substitutions and two strains with D222E substitutions. The other two strains had substitutions at 190-helix region (residues 184–191), one with L191I and the other had S190N substitutions.

### Emergence of pH1N1-HA E374K mutants at HA2 of pH1N1 related to different intervention strategies in various phases over the epidemic period

Besides the variations of antigenic sites at HA1, we identified one unique amino acid residue mutation (change from E to K) at residue 374 which is located at the stalk region of HA2 (Figure 4B,C,D). We then examined the dynamic changes of this pH1N1-HA E374K (E374K) mutant as it evolved through different epidemic periods and searched for possible associated important factors, using both univariate and multivariate analyses. The E374K mutant was first detected on August 26, 2009 (the 34^th^ week of 2009, 3 weeks before the peak of the epidemic) in Taipei and on October 5, 2009 (the 40^th^ week - one week before the peak of the epidemic) in Kaohsiung.

**Figure 5:**
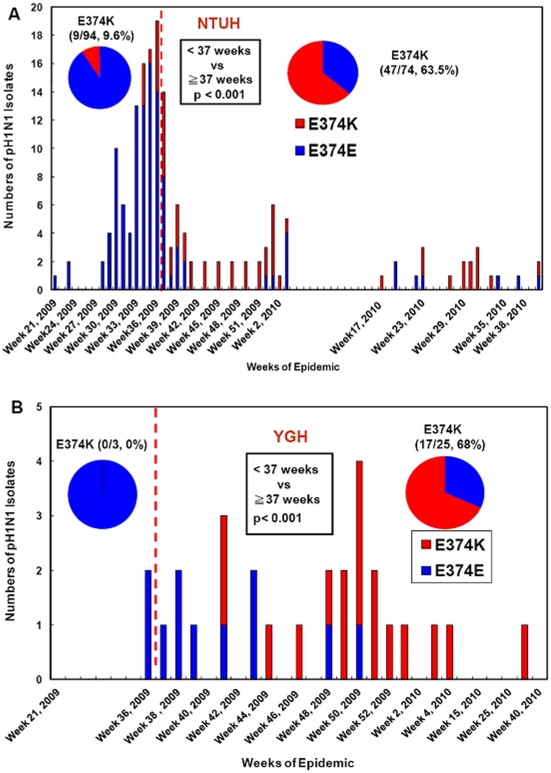
Temporal distributions of pH1N1-HA E374K mutants before and after the peak time of epidemics in Taiwan, 2009–2010. **A:** Distribution of pH1N1-HA E374 mutants isolated at NTUH (National Taiwan University Hospital). **B:** Distribution of pH1N1-HA E374 mutants isolated at YGH.

The frequency of the detected E374K mutants was significantly higher in the post-peak period of the epidemic (≧37weeks, September 13 and later, 2009) than in the pre-peak period (<37 weeks) in both Taipei [63.51% (47/74) vs 9.57% (9/94), p<0.001] and Kaohsiung [68% (17/25) vs 0% (0/3), p<0.001] (Figure 5). Taken together, the overall frequency of the identified E374K mutant was significantly higher in the post-peak period of the epidemic than those in the pre-peak period [64.65% (64/99) vs 9.28% (9/97), p<0.0001], regardless of geographical variations (northern vs southern Taiwan) (Figure 5).

Three public health measures, antiviral use, class suspensions and vaccination, which were implemented sequentially during the 2009 pandemic influenza H1N1, were analyzed in detail to examine their roles in the viral mutation. In order to understand the effect of pharmaceutical intervention on the dynamic changes of E374K, we compared the frequencies of E374K mutants isolated before versus after the initiation of nation-wide use of Oseltamivir (Tamiflu) for treating all ILI patients testing influenza A antigen-positive on August 1, 2009 (the 30^th^ week). The positive rate of pH1N1 still continuously increased and then peaked at week 37 in northern Taiwan. There was an association between Tamiflu use and subsequent emergence of E374K [before vs after initiation use:[0% (0/17) vs 40.78% (73/179), p<0.001]. However, using this antiviral drug was not associated with the emergence of other mutations in the four antigenic sites [before (29.41%, 5/17) vs after (17.83%, 28/157), p = 0.324 (Table 3)].

**Table 3:** Comparison of the percentages of the pH1N1-E374K mutants in HA2 and other variants at the four antigenic sites and their relationships with different intervention strategies in various epidemic periods, 2009–2010.

		Percentages of Mutants						
Periods	Intervention Strategies	HA2		HA1				
		Number of isolates	E374K	Number of isolates	Ca	Cb	Sa	Sb
**Before Aug. 1, 2009**	**No any intervention**	17	0 (0%)^1^	17	4* (23.3%)^3^	0 (0%)	2* (11.8%)^3^	1 (5.9%)
**Aug 1–Nov. 18, 2009**	**Start to pay Tamiflu by National health Insurance**	123	32 (26.0)%	101	9 (9.2)%	2 (2.0%)	1 (1.0%)	1 (1.0%)
**Nov.19–Dec 19, 2009**	**Implement of vaccination in 6–18 years old**	9	7 (77.8%)^2^	9	2 (22.2%)^4^	0 (0%)	0 (0%)^4^	0 (0%)
**After Dec 19, 2009**	**Post nation- wide vaccination campaign**	47	34 (72.3%)	47	4** (8.5%)	0 (0%)	11** (23.4%)	1 (2.1%)

1 . The effect of intervention of antiviral agent on the percentages of E374K mutants: Before (0%, 0/17) vs after (40.78%, 73/179), p<0.001 (Fisher's exact test).

2 . The effect of intervention of vaccination on the percentages of E374K mutants: Before (22.86%, 32/140) vs after (72.34%, 41/56), p<0.001 (Fisher's exact test).

3 . The effect of intervention of antiviral agent on the percentages of the pH1N1 variants with substitutions at four antigenic sites (Ca, Cb, Sa, Sb): Before (29.41%, 5/17) vs after (17.83%, 28/157), p = 0.324 (Fisher's exact test).

4 . The effect of intervention of vaccination on the percentages of the pH1N1 variants with substitutions at four antigenic sites (Ca, Cb, Sa, Sb): Before (11.86%, 18/118) vs after (25%, 15/56), p = 0.097 (Fisher's exact test).

*: Two strains with double mutations in Ca and Sa sites.

**: Three strains with double mutations in Ca and Sa sites.

To further investigate the relationship between temporal changes in the frequency of E374K mutants and time phases regarding the other two major public health intervention strategies, we first analyzed E374K mutants related to the time phases of class suspension intervention in Taipei City. This was because the new class suspension policy was implemented when fall semester started - about two months before the pH1N1 vaccination. The results showed that only three pH1N1 isolates (4.76%, 3/63) had E374K substitutions detected one week before the intervention of class suspension (September 1, 2009) (Table 4). When the fall semester began in early September, E374K mutant was still present and then sharply increased in the first three weeks [wks 36–38: 26.32%(5/19), 42.86%(6/14) and 66.67%(2/3), respectively], despite the nationwide class suspension policy initiated on September 1, 2009. The frequency of E374K substitutions increased throughout the epidemic, being much higher in the pH1N1 strains isolated at the peak of the 2^nd^ class suspension wave (weeks 41–45, eg. peak of schoolchildren cases) than those weeks before class suspension intervention (weeks 34 and before) [100% (6/6) vs 4.76% (3/63), p<0.001] (Table 4). We then explored E374K mutants before and after implementing vaccination program by dividing the post-peak epidemic period into three phases: (1) the peak-epidemic to pre-vaccination [Peak-Epi-Pre-Vac] phase (week 37–43) was from the peak of the epidemic (September 13, 2009) to the starting date (November. 1, 2009) of the vaccination program; (2) during implementing vaccination program [D-Vac] phase (week 44–50) from the initiation date of pH1N1 vaccination program targeting at health worker population to one week post nation-wide vaccination program (November 1-December 19, 2009), and (3) post-national vaccination [Post-Nat-Vac] phase (>week 50) was one week after the post-nation-wide vaccination campaign (after December 20, 2009). The frequency of E374K was significantly higher in the phases of “during vaccination (D-Vac)” (88.2%, 15/17) and “Post-Nat-Vac” (69.1%, 29/42) than in the time interval from peak-epidemic to pre-vaccination [Peak-Epi-Pre-Vac] phase (50%, 20/40) (p = 0.017) [Table 3,4].

**Table 4:** Univariate analysis of the factors associated with the frequency of the pH1N1 HA E374K mutants isolated in Taiwan, 2009–2010.

Factors	Numbers of isolates	E374K	Fisher's exact p-values
**A. Epidemic periods**			
**1. pre-peak period of the epidemic** * (<Wk 37, before September 13, 2009)	97	9 (9.3%)	
**2. Post-peak period of the epidemic** (≧Wk 37, September 13, 2009∼)	99	64(64.7%)	**<0.0001**
**B. Class suspension in Taipei City**			
**1. Before suspension intervention** (<Wk35, before September 1, 2009)	63	3(4.8%)	
**2. Peak of suspension wave** (Weeks 41–45; October 11–November 14,,2009)	6	6(100%)	**<0.0001**
**C. Vaccination program intervention**			
1. **Peak-epidemic to pre-vaccination** [Peak-Epi-Pre-Vac](Wk 37–43, September 13–October 31, 2009)	40	20(50%)	
2. **During vaccination campaign** [D-Vac] (Wk 44–50, November 1–December 19, 2009)	17	15(88.2%)	
3. **Post–national vaccination campaign** [Post-Nat- Vac](>Wk 50, After December 20,2009)	42	29(69.%)	**0.017**
**D. Gender** **Males**	118	41(34.8%)	
**Females**	78	32(41%)	0.451
**E. Age (Years)**			
**1. Pre-peak period of the epidemic**			
<12	45	3(6.7%)	
12–18	27	4(14.8%)	
>18	25	2(8%)	0.552
**2. Post-peak period of the epidemic**			
<12	41	29(70.7%)	
12–18	18	12(66.7%)	
>18	40	23(57.5%)	0.472
**F. Gender and Age**			
**a) Males** <12	52	21(40.4%)	
12–18	33	10(30.3%)	
>18	33	10(30.3%)	0.534
**b) Females** <12	34	11(32.4%)	
12–18	12	6(50%)	
>18	32	15(46.9%)	0.394
**G. Population density**			
**a) Pre-peak period of the epidemic**			
1) >20000/km^2^	42	9.5%(4/42)	
2) <20000/km^2^	40	7.5%(3/40)	1
**b) Post-peak period of the epidemic**			
1) >20000/km^2^	38	25(69.8%)	
2) <20000/km^2^	28	19(67.9%)	1

*: Based on the positive rate detected by RT-PCR or viral culture.

### Tempo-spatial analysis of dynamic changes of E374K mutants

**Figure 6:**
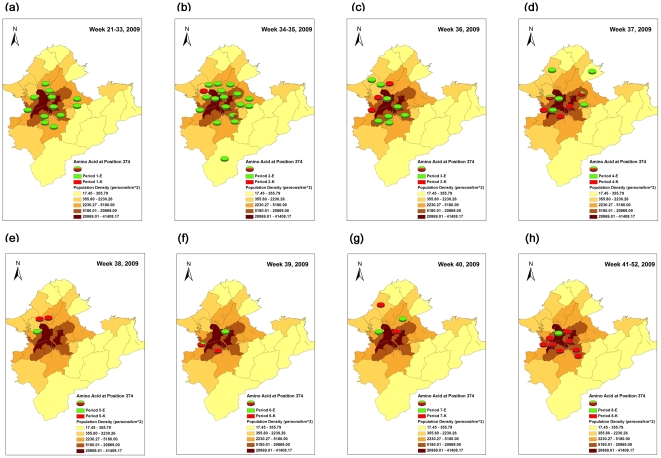
Percentages of pH1N1-HA E374K mutants of 2009 pandemic influenza A (H1N1) in Taipei metropolitan area. (a)–(h): Different time periods. Green color: virus with E374E; Red color: virus with E374K mutants; The basemap's color represented the population density.

To understand the tempo-spatial spreading of E374K mutants in different geographical areas, both global spatial autocorrelation and local cluster correlation analyses of E374K from week 21 to week 52, 2009 were performed. Using Moran's I test to assess global spatial autocorrelation clusters, we found that pH1N1 cases within weeks 41–52 showed only mild spatial clustering (Table 5, Figure 6). Furthermore, we performed local Moran's I test to check where the spatial clusters were present during weeks 41–52. The results indicated that seven districts in Taipei presented significant high-high spatial clusters (Figure S1).

**Table 5:** Global spatial autocorrelation analysis of E374K in Taipei metropolitan area, 2009.

Epidemic periods	Numbers of isolates	Global Moran's I	p-values
**Weeks 21–33**	37	Control	
**Weeks 34–36**	48	−0.05327	0.451
**Weeks 37**	14	−0.01657	0.836
**Weeks 38–40**	12	0.00864	0.404
**Weeks 41–52**	10	0.16498	**0.000004**

Besides temporal factors and spatial clusters, univariate analysis on other factors including gender, age groups and district-specific population densities did not show any significant differences in the frequency distribution of E374K mutant stratified by pre-peak and post-peak periods of the 2009 epidemic (Table 4).

To further identify the role of factors that might simultaneously affect the dynamic changes of E374K mutants, we used multivariate logistic regression models considering the variables of age groups (0–4.9, 5–14.9, 15 and over 15 years old), time periods (weeks), population density, and spatial clustering. The results indicated that only the time periods and spatial clustering showed higher odds ratios (OR) [time periods: OR = 1.529, p<0.001, spatial cluster: OR = 4.565, p = 0.047] after controlling for the two most important confounders, age and population density (Table 6).

**Table 6:** Odds ratios for the pH1N1-HA E374K mutants isolated in Taipei City by multivariate logistic regression analysis.

Factors	Adjusted Odds Ratios (OR) for Mutant E374K versus Wild type E374E	95% CI of OR	p-values
**Time**			
Weeks of the 2009	1.5	1.3, 1.9	**<0.001** *
**Spatial Cluster**			
Non-cluster Districts	1		
Cluster Districts	4.6	1.0, 20.4	**0.047** *
**Age (Yers)**			
≧15	1		
0–4.9	0.1	0.0, 4.0	0.224
5–14.9	1.8	0.6, 5.5	0.338
**Population density**			
Low (<20455/km^2^)	1		
High (>20455/km^2^)	1.4	0.4, 5.5	0.610

Abbreviation : CI, confidence interval; OR: Odds ratio.

*p<0.05.

### Global comparison on the temporal distributions of pH1N1-HA-E374K Mutants in different influenza transmission zones in 2009

In order to compare the dynamic changes of E374K in Taiwan with those in other countries, we collected nucleotide sequences of 1462 strains of the 2009 pH1N1 from the NCBI Influenza Virus Resource and analyzed. The monthly distributions of E374K in the five WHO influenza transmission zones [29] were shown in Figure S2. The results indicated that the E374K mutant steadily increased in Taiwan from August to December, 2009 and became the major circulated strains close to December in Taiwan [84.62% (11/13)] and in other Asia countries [50% (7/14)]. Although E374K mutants were detected earlier in America and Europe (July, 2009) than in Taiwan (August, 2009), their frequencies were unstable with fluctuation in the following months without being fixed early.

### Effect of the E374K mutation on the antigenicity and neutralization activity of HA

To understand the possible effect of an E374K mutation located at the stalk of HA2 in the cavity where the fusion domain of mature HA molecules [30], [31] might have an impact on the antigenicity or neutralization activity of pH1N1, we conducted cross-antibody tests, using the same paired patients' serum specimens and virus isolates. Two convalescent serum samples collected from pH1N1 infected patients, one with wild type viruses (E374E) and one with the mutant strain (E374K), were employed for measuring the levels of antibody responses against both strains simultaneously, using cross HI and MNt tests. By applying Archetti and Horsfall's formula [32] for the calculation, the antigenic difference between the two tested virus strains (when both viruses and their antisera were compared in cross tests) represented by the r values for HI and MNt tests, r values of 1.414 and 1, respectively, were generated. An r value of 1 means “antigenic identity”. The larger the r value, the less antigenic relatedness of the two compared virus strains is obtained. In general, an r value of 4 or greater indicates a significant antigenic difference between the two tested strains. Table S1 shows that there was no significant antigenic distance in the antibody levels measured by HI and MNt tests between the pH1N1 wild-type (E374E) and E374K mutant strains. We then increased sample size to test six pH1N1 strains using six children's serum samples with higher serotiters (≥1∶640) for increasing the sensitivity, the results still showed there were no significant differences in HI serological reactions between the wild-type E374E and the E374K (Table 2). This finding was also confirmed by using the pH1N1-immunized sheep serum as the reference antibody for HI (Table 2).

### Investigating the role of pH1N1 virus HA1 variants in clinical severity

In order to know if the possible association between amino acid residue substitutions at D222G/E (located at antigenic site Ca) or temporal increases in frequencies of Q293H in pH1N1 viruses with clinical severity documented in other countries [15], [17], [18], [33]–[35] might also be present in Taiwanese pH1N1 isolates, we compared the distribution of amino acid substitutions of pH1N1 isolated from patients with mild ILI symptoms (116 cases) versus those from patients with severe complications (52 cases). To our surprise, there was no significant difference in the frequency of pH1N1 with D222G/E or Q293H mutations isolated between ILI patients with mild and severe complications (Table S2). In addition, there was no significant association between E374K mutants or other pH1N1 new variants (N125D, S203T, R205K) and the severity of clinical outcomes (Table S2). The other mutations of HA D131E and S186P variants found conferring viral virulence of A/California/04/2009 pH1N1 adapted in mice [36], were also not found during our study period.

### Analysis of the diversity of amino acid residues of NA in Taiwanese pH1N1 isolates

Since NA is important for virus release and assisting efficient replication of influenza virus [8]–[10], NA nucleotides (1410 nucleotides) from 40 available strains of pH1N1 virus isolated in Taiwan were collected for analyzing the dynamic changes of amino acids in the NA. Using the vaccine strain A/California/7/2009 as a reference, the identities of nucleotides and amino acids were found to be 99.55% and 99.39% respectively. The average substitution rates of nucleotides and amino acids were 4.6.×10^−3^ per nucleotide per gene and 6.26.×10^−3^ per amino acid per protein. The differences in the numbers of amino acid changes of NA ranged from 2–6. Similar to HA, the tendency of increasing frequency of more cumulative numbers of amino acid mutations in NA over different epidemic time periods was also observed [post-peak period of the epidemic (61.90%, 13/21) versus pre-peak period of the epidemic (36.84%, 7/19)], though the pH1N1 with cumulative number of amino acids ≥3 was not significantly different between pre- and post–peak epidemic periods (p = 0.205) (Table 1). In addition, all 40 isolates showed two mutations of V106I and N248D (number from initiation codon of NA) (Table S3). Besides these two most frequent residues changes, other sporadic residues substitutions, including amino acids 4, 10, 11, 16, 19, 34, 66, 82, 84, 119, 166, 188, 189, 242, 309, 329, 365, 381, 382, 394, 416, 426, 435, 448, 452, 453, 462 and 468, also occurred in certain isolates (52.5%, 21/40). Two mutants are worth mentioning: (1) only one strain (A/NTU340/2010) had an E119K mutation (2.5%, 1/40) located at the catalytic site of NA and (2) another strain (A/NTU389/2010) had N329I mutation (2.5%, 1/40) located at an antigenic site [37].

Two strains of all the 40 sequenced Taiwanese pH1N1isolates had co-mutation in the HA and NA regions. One strain, A/NTU4223/2009 isolated from a mild case on July 15,2009 had co-substitutions in HA (S203T, D222G) and NA (V106I, V166I, N248D), and the other strain, A/NTU340/2010 isolated from a pneumonia case on January 19, 2010 had co-substitutions in HA (S203T, K163E) and NA (I34T, V106I, E119K, N248D, G382E, D416N). However, these two co-mutations of HA and NA pH1N1 did not persist in the human population.

Most importantly, none of the 40 analyzed pH1N1 strains had the H275Y mutation, a site of resistance to oseltamivir.

### Analysis of the changes at N-glycosylation sites of HA and NA

Since the addition of the N-glycosylation site in the globular head of HA might provide influenza virus with the ability to evade antibody pressure [38], [39], we further analyzed the possible changes in N-glycosylation sites from Taiwan's 78 strains of pH1N1 collected from June 11, 2009 to August 2, 2010 (using A/California/07/2009 as the reference strain). The results show that only two strains, A/NTU8112/2009 and A/NTU11/2010 isolated at post-peak epidemic period had lost one N-glycosylation site of HA at residue 11–13 and 23–25, respectively. All other 76 strains of pH1N1 strains had the conserved seven N-glycoslation sites (residues 10, 11, 25, 87, 276, 287 and 481) of HA. In contrast, all eight N-glycoslation sites (50, 58, 63, 68, 88, 146, 235 and 386) of NA were retained in the 40 sequenced strains.

### Summary of pH1N1 Variants in HA and NA

In summary, we identified three fixed mutants at HA and NA of pH1N1, including (1) the double mutants of NA (V106I and N248D) that occurred in foreign countries with 0% of wild-type, (2) S203T mutants also emerged in overseas but with about 5% of wild-type detected in Taiwan, and (3) E374K started from 0% but progressively increased and finally replaced most wild-type pH1N1.

## Discussion

Newly emerging influenza pandemics provide the best opportunity to follow the dynamic changes of viral mutants in the initial phase as well as after public health measures have been instituted. We performed such studies in two high-density metropolitan areas in Taiwan, and made four observations that may enhance our understanding of influenza epidemiology. First, a significantly higher cumulative number of amino acid changes in HA and NA was found in the post-peak period of the epidemic. Second, a pH1N1 mutant with a unique change E374K in HA2 detected first at 34^th^ week in Taipei and 6 weeks later in Kaohsiung, survived more successfully than other variants through transmission chains and became the major circulating strains in the post-peak period. Such well-adapted mutants were favored in certain areas of metropolitan Taipei, where pH1N1 cases had significantly higher spatial clusters during the peak of the second epidemic wave (weeks 40–47). Third, tempo-spatial increases in E374K for six weeks matched well with the second wave of class suspension (41–45^th^ weeks). These increasingly dominant mutants persistently circulated for an additional three weeks before the first day of vaccination for healthcare workers and continued for two more weeks before implementing mass-vaccination for schoolchildren. Fourth, vaccination sharply reduced pH1N1 cases but at 10 months post-vaccination there was still inadequate antibody pressure to drive antigenic drifts in the circulating new dominant mutants. These findings imply that continuous selection of pandemic influenza viruses occurs most frequently at the time periods and places where numerous transmission events are happening and stress the importance of prevention measures to reduce the public health threat.

The higher frequency of cumulative amino acid changes of HA after the epidemic peak identified in this study can be explained by several factors. Due to lack of proofreading of viral RNA polymerase activity, HA has a very high rate of spontaneous mutation (estimated at 2×10^−3^ base substitutions/position per virus generation) [40]–[42]. The quasispecies of pH1N1 virus within an individual host [43]–[45] indicate that the intra-host selection of unique variants in viral populations should occur and subsequently be transmitted [46]. This natural process leads to selective advantage variants that continue to circulate in the human population [42], [47]–[48]. Additionally, the recombination of influenza virus strains within an individual (e.g. intra-host) is also likely to affect host selection. This might drive the mutated virus variants with higher replication, better fitness and more efficient transmissibility to have a selection advantage and become the dominant variant [42].

The phenotypic variations in HA with public health significance involve five major dimensions: (1) RBS important for viral entry, (2) antigenic variations, (3) glycosylation sites related to viral virulence and immune escape, (4) clinical severity, and (5) increasing epidemiological significance. Most pH1N1 viruses with one or two amino acid residue changes in the antigenic sites of HA isolated in Taiwan occurred sporadically and were not fixed during the 2009–2010 epidemic. In the RBS of pH1N1 HA, the finding of a substitution at the 220-loop region (D222E, D222G), particularly D222G, reported to be associated with clinical severity [15], [17], [18], [26], [33]–[35], [49], was not supported by our results or other reports [50]–[54]. Other RBS mutant such as I216L, which led to efficient airborne viral transmission in ferrets [55], was also not found in our study.

Antigenic variation can involve changes in antigenic sites and glycosylation sites. N125D located at antigenic site Sa of HA, which was first detected on June 6, 2010 (week 23) (e.g. late epidemic phase) and circulated through October 2010. All nine Taiwanese N125D mutants had co-existing E374K mutations, consistent with the findings from Singapore, Australia and New Zealand [56]. This N125D antigenic variant, similar to E374K, appeared after the epidemic peak but with lower frequency, indicating it had insufficient high immunological pressure to be fixed. Furthermore, HI serological titers showed no significant differences between these two strains with double mutants (HA: N125D and E374K) and the other two strains without double mutants, using pH1N1 vaccinated human serum and immune sheep serum. Moreover, the other substitutions located at Ca site (K142R, G170R, S203T and D222G) also showed no antigenic changes by HI reaction. These results imply that pH1N1 variants observed at the beginning of pandemic period had not developed the capability to escape from immune pressure, regardless of the cumulative number of amino acid changes identified at the antigenic sites. In addition, we did not detect any new glycosylation sites in the HA [57], [58]. Taken together, the lower numbers of cumulative amino acid changes, the less frequent multiple mutations at four antigenic sites and the lack of additional glycosylation sites identified in this study support the evidence of insufficient selection pressure. Immune selection pressure has been the main driving force for antigenic drift of human seasonal influenza viruses [59]–[62]. This was not observed from the beginning pandemic period in 2009 till ten months after vaccination.

Our results in Taiwan did not find the three pH1N1 variants, Q293H, D131E, and S186P, that were associated with clinical severity. Such an inconsistent finding is likely due to mass application of antiviral and other public health prevention measures implemented in Taiwan to reduce viral transmissibility. This implies that clinically severe cases might have emerged under special epidemiological settings and searching for these answers will be helpful for informing future public health prevention decisions.

After analyzing all pH1N1 variants throughout the epidemic, this study identified two fixed mutation changes (S203T and E374K) in HA with epidemiological significance. However, these two mutations were not significantly co-varied (Fisher's exact p = 0.08). The overall high percentage (97.13%, 169/174) of total Taiwanese pH1N1 viruses which had the S203T substitutions is consistent with other reports [54], [63]–[67]. These results indicate that such substitutions might have occurred before their introduction to Taiwan. Interestingly, this small change in a side chain near the monomer-monomer interface appears not to have had a dramatic effect on the structure of HA [27], nor on HI titers. In contrast to S203T, the E374K mutation occurred throughout the epidemic in Taiwan. Application of Tamiflu, that proved to reduce viral load [68], [69], was implemented for all influenza A antigen-positive ILI patients from Aug 1, 2009 (week 30) and this might have delayed the peak of epidemic before the vaccination program [70]. When the fall semester began in early September, the E374K mutant sharply increased in the first three weeks despite the nationwide class suspension policy initiated on September 1, 2009. After week 37, pH1N1 variants strikingly increased the number of amino acid substitutions to five or more and elevated the percentage of E374K variant replacing E374E. The class suspension did not stop the transmission of this E374K mutant, while the percentages of E374K mutant in the first wave (wks 35–40) and second wave (wks 41–45) of class suspension in Taipei City were 30.16% (19/63) and 100% (6/6) respectively. The increased transmission of pH1N1 might provide more chances to spread E374K mutant in the human population. The period of high percentage of E374K mutant matched well with the period before the start of mass vaccination of pH1N1 in elementary and high schools on November 16, 2009.

The tempo-spatial epidemiological conditions facilitating the increasing dominance of E374K mutants even before vaccination, supported by spatial epidemiology and multivariate analyses, were the higher spatial clusters of E374K mutants occurring in the post-peak of the epidemic period and significantly high spatial clustering in seven district regions with greater population densities. Because of the limited number of Taiwanese pH1N1 viruses (n = 121) captured in this study, it may be difficult to fully characterize the impact of the tempo-spatial effects on E374K mutation. A larger sample size is needed to verify this conclusion.

For the newly emerged pH1N1, most of the persons born after 1957 were susceptible to this virus [71], [72]. A lesser level of herd immunity, that may be insufficient to result in high immune selection pressure, explains why less substitution of amino acids and less percentage of E374K mutants were isolated in the early pre-peak period of the epidemic. In fact, the overall vaccine coverage rates of pH1N1 in all Taiwan areas and Taipei by January 29, 2010 were about 24.3%, and 21.8%, respectively [22]. The level of community-based herd immunity plus the extremely high vaccine coverage (74.7%, 271460/363403, January 29, 2010) in Taipei's 7–18 year old school children did reduce the size of the pH1N1 epidemic and transmission opportunities. Therefore, even at ten months after the nationwide vaccine campaign, there was insufficient selection pressure for antigenic drift of pH1N1 in Taipei. Taken together, all these results strongly support that E374K widely spread in areas with day-to-day schoolchildren gatherings even during the period of class suspension and anti-viral programs. It is very likely that unhygienic behavior, the high-density public transportation system, crowding, or all of the above might contribute to the tempo-spatial clustering of E374K mutants. Thus, the viral population size of the E374K mutant increased through epidemics, particularly at the pre-vaccination phase during post-peak periods. Certainly, without anti-viral agents, class suspension, and vaccination, more virus circulation and diversities of mutants may have emerged. On the other hand, the tempo-spatial epidemiological conditions at peaks might facilitate the selection of more virulent viral variants and thus confer increasing epidemic severity in later waves with more fatalities. This happened as documented in past influenza pandemics [73]–[75] and other viral pandemics as well [76], [77], once public health interventions had not been timely effective. These findings indicate that more attention **s**hould be paid to non-pharmaceutical measures [78], [79] for future novel influenza viruses with pandemic potential.

Global comparison of the evolution of the E374K mutations from June to December of 2009 [19], [26], [53], [63], [64] found that lower numbers of cases with E374K mutation were reported in Canada (3.4%, 8/235) and Finland (2.38%, 3/126) in the temperate zone but more cases with this mutation were reported in Taiwan (37.24%, 73/196) and Singapore [September (14%), October (28%), November (55%)] in the subtropical and tropical zones with high population density, respectively [19], [53], [64]. The roles of climate, population density, and human-to-human contact behaviors in facilitating the fixing of E374K mutants in those particular regions have not been clarified, indicating that timely international collaboration is needed among countries in different climate zones with various epidemiological characteristics once novel influenza virus emerges in future years.

The virological mechanism that might explain the fitness of the E374K mutant in the viral population is worth pursuing. Among several pH1N1 variants, E374K variants were not evident in the early epidemic period and their fitness would be random due to the lack of consistent co-substitutions [62], [80]. Single residue deletions in the HA fusion peptide can lead to loss of membrane fusion activity [81], however, the first 24 N-terminal residues of HA2 were conserved in all the 78 Taiwanese pH1N1 isolates. No significant differences in cross-MNt and HI serologic reactions were observed between the wild-type E374E and the E374K mutant, even using pH1N1-positive human and sheep serum samples with high titers. More results are needed with serum samples from patients experienced the 1918 influenza pandemic or with higher B-cell memory responses to clarify the possible mechanism of immune selection of this mutant, particularly because the fusion portion of HA2 offers partial protection for heterologous neutralization [82]. Although E374K mutants had the similar growth capability in MDCK cells (Figure S3), other biological characteristics that might be changed through affecting the stability between oligomers interaction [19], [27], [53] or be less recognized or cleared by immune system facilitating persistent transmission in human population [83] need further investigation.

In conclusion, this is the first study examining dynamic changes of pH1N1 viruses through integrating virological surveillance, tempo-spatial epidemiological characteristics, public health interventions, clinical severity and serological findings, hopefully providing fundamental information during the pandemic. This study had selection bias in the hospitals, patients' giving specimens, and fatal cases. A prospective large cohort study that can integrate with a high quality virological surveillance system through collecting clinical, epidemiological and intervention attributes, and meteorological variables plus deep sequencing analysis [84]–[86] of virus population random directly from the ILI patients, will provide the best understanding on the micro- and macro-mechanisms of viral selection and fitness. Future efforts should identify the biological significance and explore the mechanisms involved in the fitness of pH1N1 viruses with amino acid residue substitutions in RBS sites, antigenic sites and the fusion regions of HA and their phenotypic characterization with human respiratory cell lines [87]. Moreover, other segments of the viral genome, such as NS1 [88] and polymerase genes [89], might impact mutant fitness, and requires a more detailed investigation. Certainly, real-time comparative studies on the relationship between intervention strategies and molecular changes of pandemic influenza virus through international collaboration will be an important step for global control of influenza.

## Supporting Information

Figure S1 **Spatial analysis of the laboratory-confirmed pH1N1 cases in Taipei City by local indicators of spatial association (LISA).**
(TIF)

Figure S2 **Global dynamic monthly distributions of pH1N1-HA E374K mutants in 2009.** Monthly percentages of pH1N1-HA E374K mutants in Taiwan were compared with those in the five WHO influenza transmission zones. The E374K was absent from African and Oceania in 2009.
(TIF)

Figure S3 **The growth yields of the pH1N1-HA-E374K (mutant) and the pH1N1-HA-E374E (wild type) in MDCK cells.** The cells were infected with viruses at 0.01 multiplicity of infection (MOI) and harvested at different time points of post-infection. The virus yields in the culture medium were determined by real-time PCR.
(TIF)

Table S1 Serotiters of antibody responses between wild-type E374E and E374K mutant viruses of the pH1N1 measured by HI and micro-Nt Tests, using their respective serum samples directly obtained from the two patients isolated in December, 2009.
(DOC)

Table S2 Investigating on the association between amino acid residue changes in the HA of Taiwanese pH1N1 viruses and clinical severity.
(DOC)

Table S3 Dynamic of amino acid changes of the pH1N1-NA isolated in Taiwan, 2009–2010.
(DOC)
