# Supplementary figures and images for: Emerged HA and NA Mutants of the Pandemic Influenza H1N1 Viruses with Increasing Epidemiological Significance in Taipei and Kaohsiung, Taiwan, 2009–10
Source: PLoS One. 2012 Feb 6;7(2):e31162. doi: 10.1371/journal.pone.0031162 (PMC3273476; doi:10.1371/journal.pone.0031162)

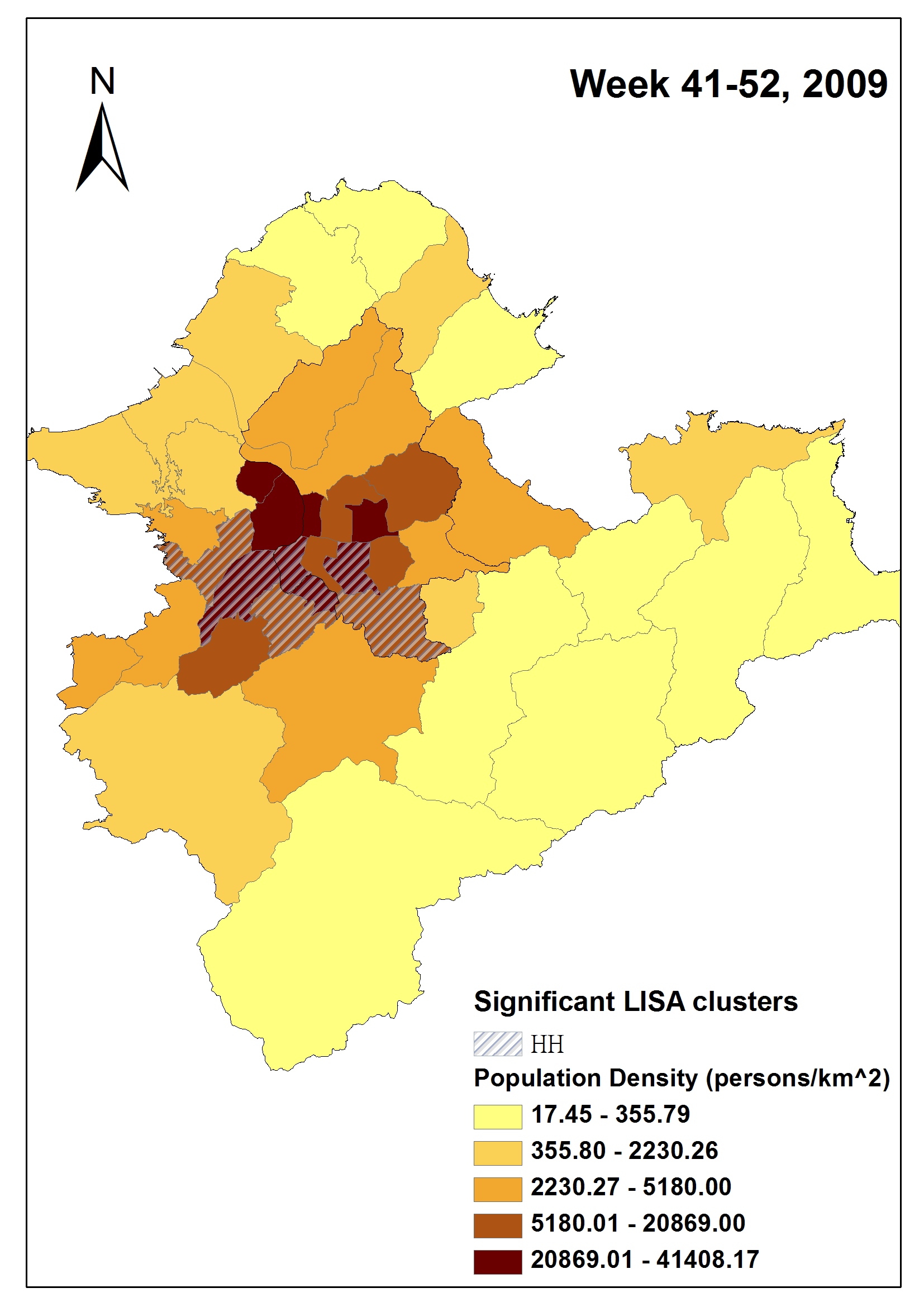

Supplement: Figure S1 — Spatial analysis of the laboratory-confirmed pH1N1 cases in Taipei City by local indicators of spatial association (LISA). (TIF) [file pone.0031162.s001.tif]

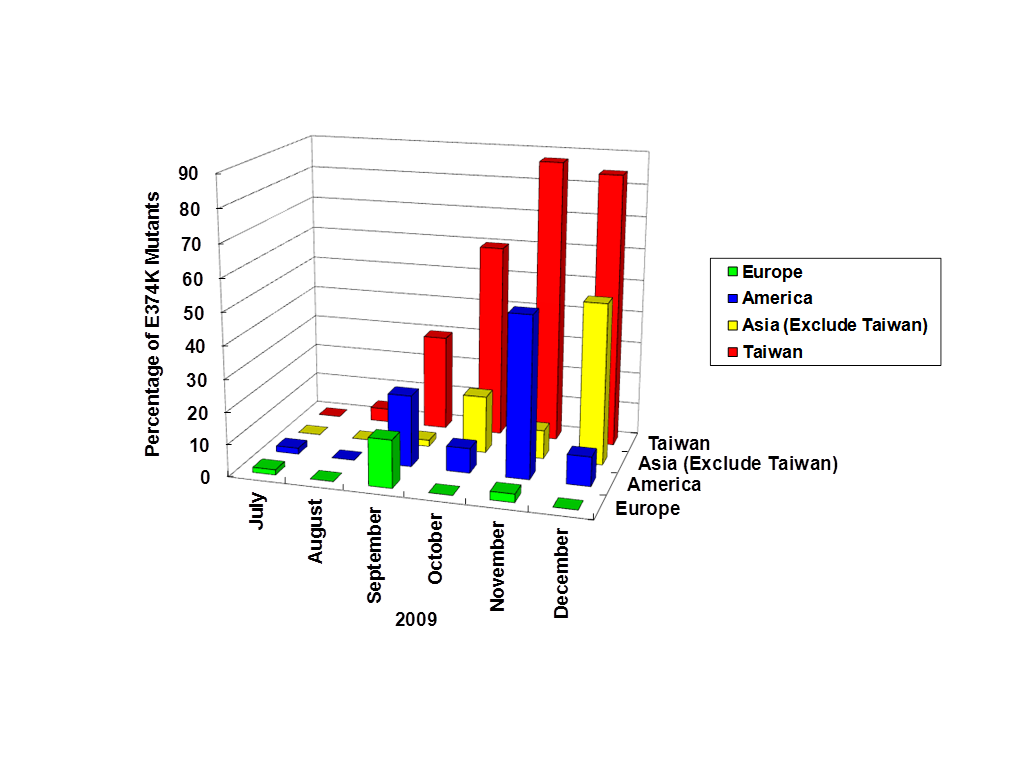

Supplement: Figure S2 — Global dynamic monthly distributions of pH1N1-HA E374K mutants in 2009. Monthly percentages of pH1N1-HA E374K mutants in Taiwan were compared with those in the five WHO influenza transmission zones. The E374K was absent from African and Oceania in 2009. (TIF) [file pone.0031162.s002.tif]

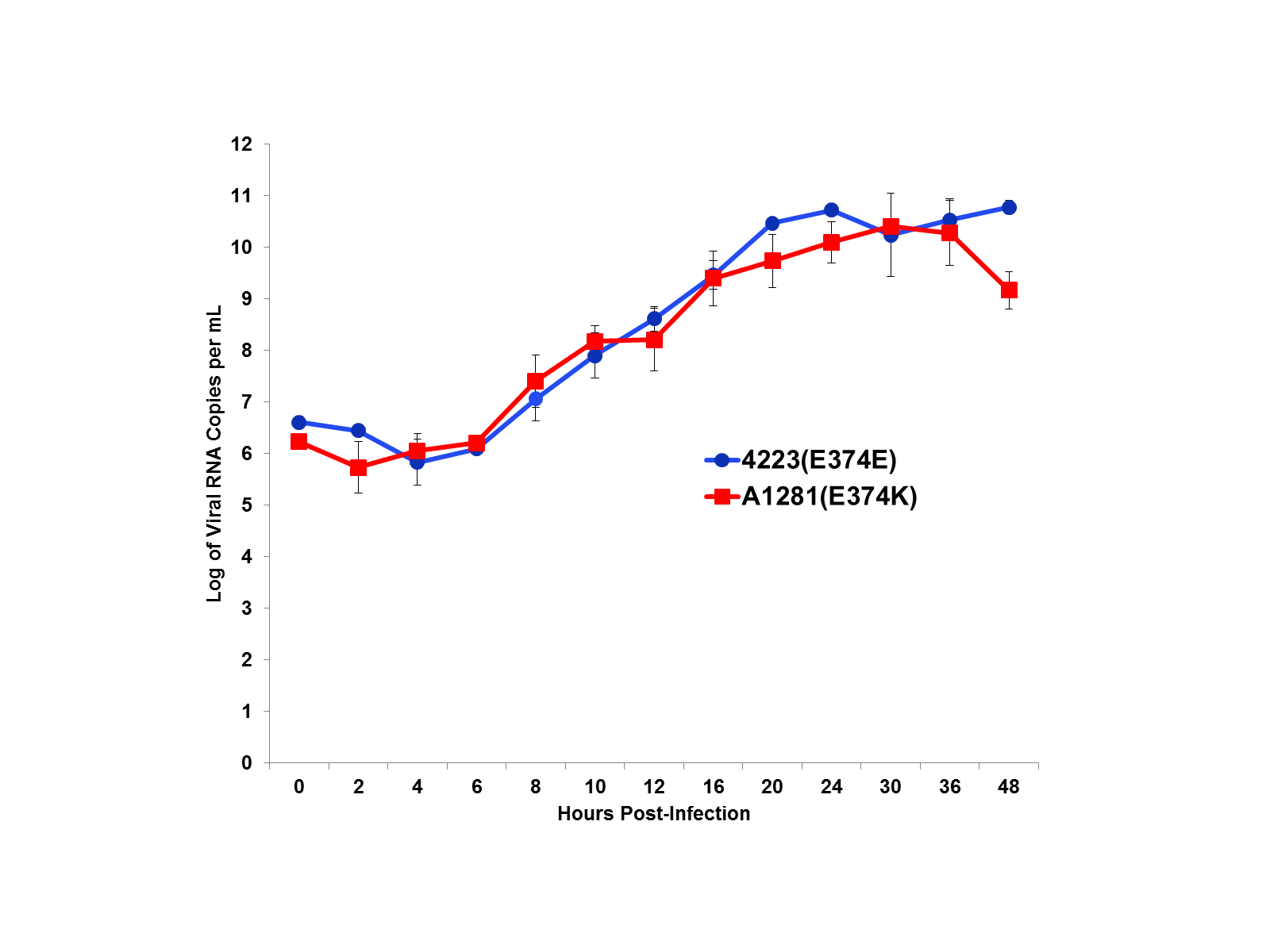

Supplement: Figure S3 — The growth yields of the pH1N1-HA-E374K (mutant) and the pH1N1-HA-E374E (wild type) in MDCK cells. The cells were infected with viruses at 0.01 multiplicity of infection (MOI) and harvested at different time points of post-infection. The virus yields in the culture medium were determined by real-time PCR. (TIF) [file pone.0031162.s003.tif]
