# Supplementary material for: Emerged HA and NA Mutants of the Pandemic Influenza H1N1 Viruses with Increasing Epidemiological Significance in Taipei and Kaohsiung, Taiwan, 2009–10
Source: PLoS One. 2012 Feb 6;7(2):e31162. doi: 10.1371/journal.pone.0031162 (PMC3273476; doi:10.1371/journal.pone.0031162)
Supplement: Table S1 — Serotiters of antibody responses between wild-type E374E and E374K mutant viruses of the pH1N1 measured by HI and micro-Nt Tests, using their respective serum samples directly obtained from the two patients isolated in December, 2009. (DOC) [file pone.0031162.s004.doc]

**Table S1.** Serotiters of antibody responses between wild-type E374E and E374K mutant viruses of the pH1N1 measured by HI and micro-Nt Tests, using their respective serum samples directly obtained from the two patients isolated in December, 2009.

| **Virus strains of Taiwanese pH1N1** | **YGH ID#507 (E374E) serum** | | **Y GH ID#724(E374K) serum** | |
| --- | --- | --- | --- | --- |
|  | **HI** | **Micro-Nt** | **HI** | **Micro-Nt** |
| **YGH ID# 507(E374E)** | 640 | 640 | 640 | 640 |
| **YGH ID# 724(E374K)** | 320 | 640 | 640 | 640 |
