# Supplementary material for: Emerged HA and NA Mutants of the Pandemic Influenza H1N1 Viruses with Increasing Epidemiological Significance in Taipei and Kaohsiung, Taiwan, 2009–10
Source: PLoS One. 2012 Feb 6;7(2):e31162. doi: 10.1371/journal.pone.0031162 (PMC3273476; doi:10.1371/journal.pone.0031162)
Supplement: Table S2 — Investigating on the association between amino acid residue changes in the HA of Taiwanese pH1N1 viruses and clinical severity. (DOC) [file pone.0031162.s005.doc]

**Table S2.** Investigating on the association between amino acid residue changes in the HA of Taiwanese pH1N1 viruses and clinical severity.

| **Amino acid changes at HA region** | **Clinical Outcomes** | | |
| --- | --- | --- | --- |
|  | **Mild cases (116 cases)** | **Severe cases* (52 cases)** | **Fisher’s exact p-values** |
| **Q293G** | 98.3%(114/116) | 98.1%(51/52) |  |
| **Q293H** | 1.7%(2/116) | 1.9%(1/52) | 1 |
|  |  |  |  |
| **D222D** | 97.4%(113/116) | 98.1% (51/52) |  |
| **D222G** | 1.7% (2/116) | 0% (0/52) | 1 |
| **D222E** | 0.9% (1/116) | 1.9% (1/52) | 0.530 |
|  |  |  |  |
| **S203S** | 3.5% (4/116) | 1.9% (1/52) |  |
| **S203T** | 96.6%(112/116) | 98.1% (51/52) | 1 |
|  |  |  |  |
| **N125N** | 94%(109/116) | 98.1% (51/52) |  |
| **N125D** | 6% (7/116) | 1.9% (1/52) | 0.437 |
|  |  |  |  |
| **R205R** | 94.8%(110/116) | 100% (52/52) |  |
| **R205K** | 5.2% (6/116) | 0% (0/52) | 0.179 |
|  |  |  |  |
| **E374E** | 65.2% (79/116) | 62.8% (33/52) |  |
| **E374K** | 34.8% (37/116) | 37.3% (19/52) | 0.597 |

*: ILI cases with severe complications involve any one of the following clinical manifestations within 4 weeks after disease onset: pulmonary complications that required hospitalization, neurological complications, myocarditis or pericariditis, invasive bacterial infection or intensive care unit admission
