# Supplementary material for: Emerged HA and NA Mutants of the Pandemic Influenza H1N1 Viruses with Increasing Epidemiological Significance in Taipei and Kaohsiung, Taiwan, 2009–10
Source: PLoS One. 2012 Feb 6;7(2):e31162. doi: 10.1371/journal.pone.0031162 (PMC3273476; doi:10.1371/journal.pone.0031162)
Supplement: Table S3 — Dynamic of amino acid changes of the pH1N1-NA isolated in Taiwan, 2009–2010. (DOC) [file pone.0031162.s006.doc]

**Table S3.** Dynamic of amino acid changes of the pH1N1-NA isolated in

Taiwan, 2009-2010.
